# Data in support of the negative influence of divalent cations on (−)-epigallocatechin-3-gallate (EGCG)-mediated inhibition of matrix metalloproteinase-2 (MMP-2)

**DOI:** 10.1016/j.dib.2015.12.028

**Published:** 2015-12-31

**Authors:** Gauri Deb, Sahil Batra, Anil M. Limaye

**Affiliations:** Department of Biosciences and Bioengineering, Indian Institute of Technology Guwahati, Guwahati 781039, Assam, India

## Abstract

In this data article we have provided evidence for the negative influence of divalent cations on (−)‐epigallocatechin-3-gallate (EGCG)-mediated inhibition of matrix metalloproteinase-2 (MMP-2) activity in cell-free experiments. Chelating agents, such as EDTA and sodium citrate alone, did not affect MMP-2 activity. While EDTA enhanced, excess of divalent cations interfered with EGCG-mediated inhibition of MMP-2.

## Specifications table

**Table:** 

Subject area	Biology

More specific subject area	Matrix metalloproteinases, tea polyphenols
Type of data	Figures, bar graphs
How data was acquired	Enzyme assays
Data format	Processed
Experimental factors	Treatment with EGCG and chelating agents
Experimental features	MMP-2 activity in HT1080 conditioned medium or in purified form, studied using gelatin zymography and FRET-peptide-based fluorogenic substrate assays.
Data source location	Guwahati, Assam, India
Data accessibility	All data presented in this article

## Value of the data

• Data for the first time show the effect of divalent cations on EGCG action.
• Data will help investigators to evaluate the results of their earlier experiments involving EGCG and perfectly design biochemical assays in future.
• Relevant for researchers investigating the bioavailability and therapeutic efficacy of EGCG.

## Data

**Fig. 1:**
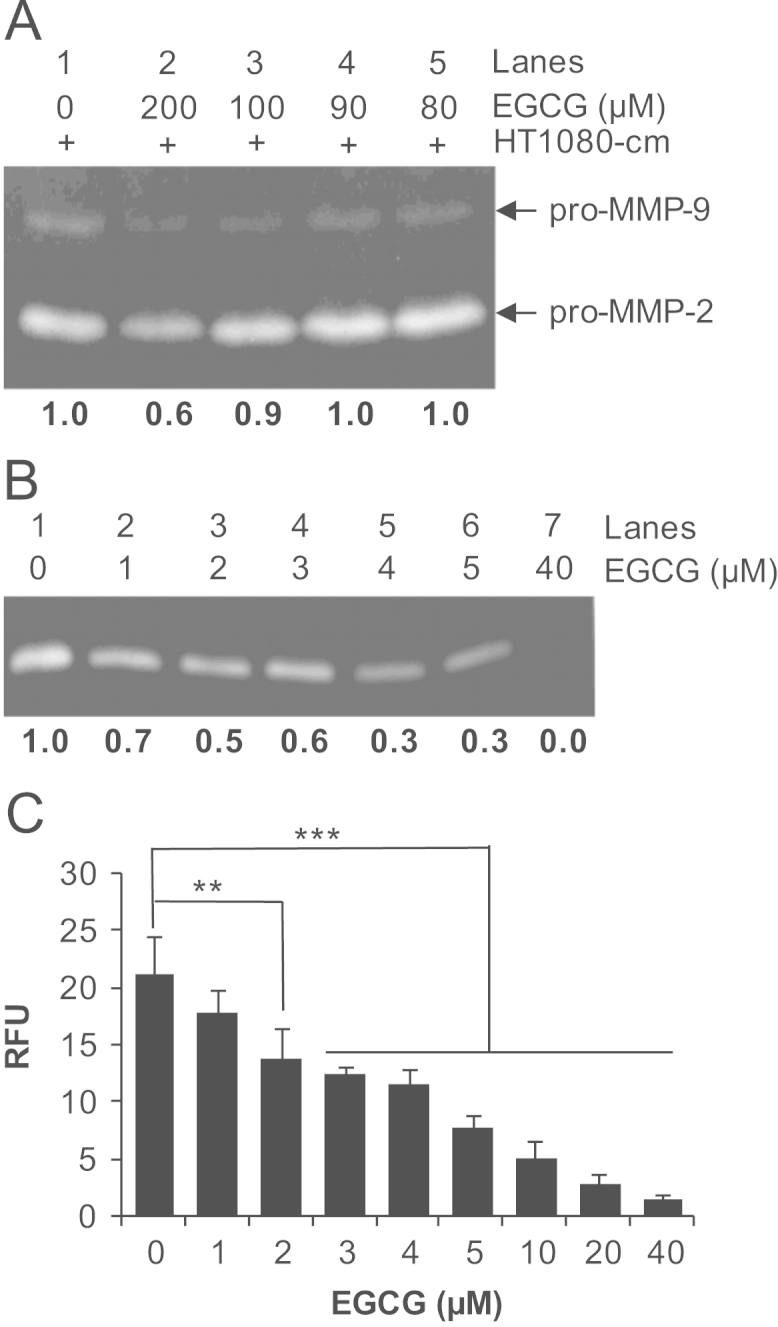
Inhibition of MMP-2 activity by EGCG. (A) Aliquots of HT1080-cm were incubated with indicated concentrations of EGCG for 45 min at 37 °C and analyzed by gelatin zymography. pro-MMP-2 and pro-MMP-9 activities are indicated by arrows. The image was analyzed by ImageJ software. The background subtracted integrated density for the MMP-2 band in the control (0 μM EGCG) was assigned a value of 1 and those obtained for others were expressed relative to control. (B) 10 ng of purified active MMP-2 was incubated with indicated concentrations of EGCG in PBS at 37 °C for 45 min followed by gelatin zymography. The numbers below each lane in the zymograms shown in A and B are relative background-subtracted integrated densities of MMP-2 bands. (C) Result of the FRET-peptide based fluorogenic substrate assay to determine the effect of EGCG on MMP-2 activity. 10 ng of purified active MMP-2 was incubated with indicated concentrations of EGCG in the assay buffer at 37 °C for 1 h followed by assaying for MMP-2 activity based on cleavage of 20 μM MMP-2 specific fluorogenic peptide substrate. RFU – relative fluorescence units. Bars represent mean±SD (*n*=3). **p<0.01, ***p<0.001.

**Fig. 2:**
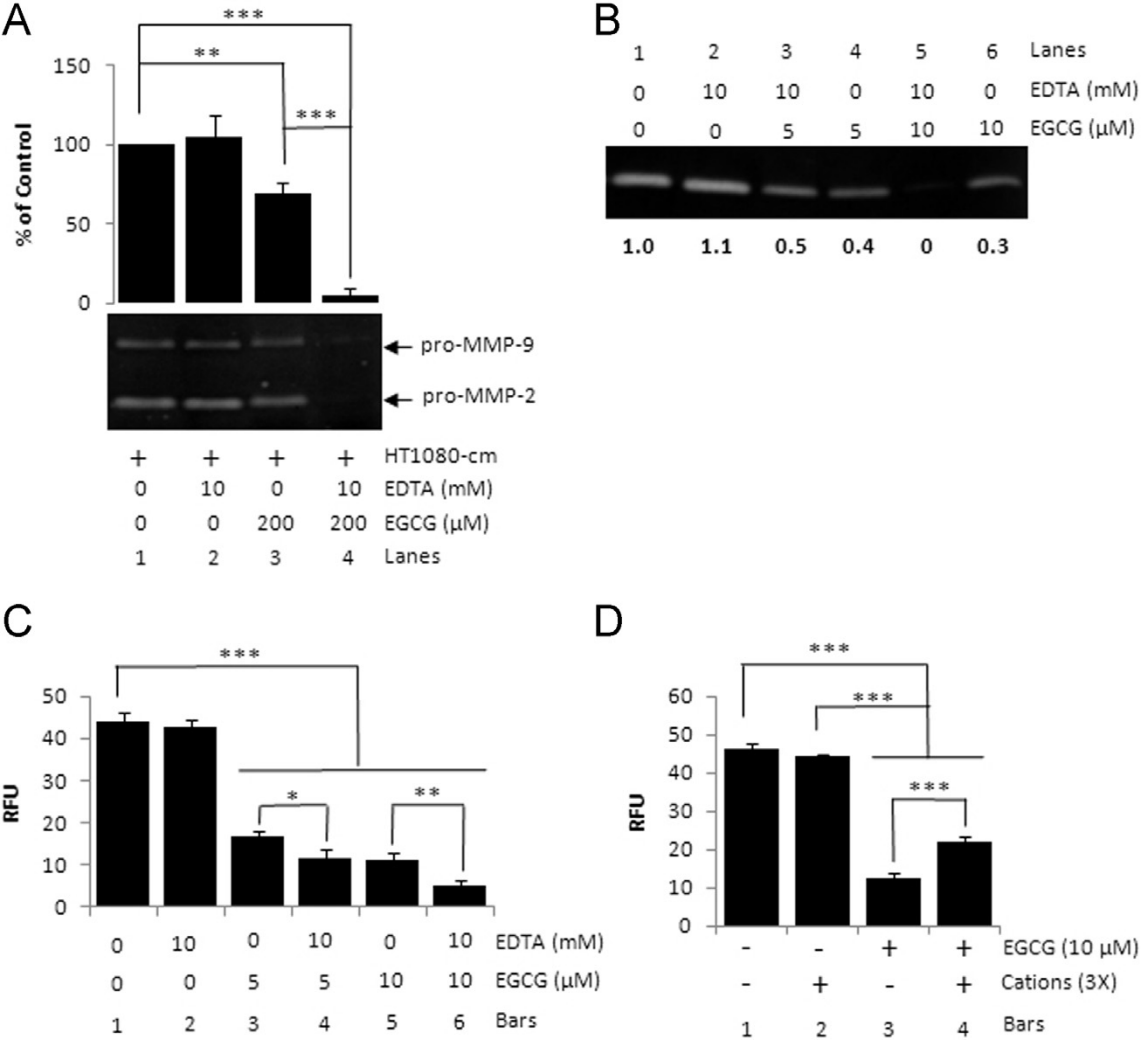
Effect of divalent cations on inhibition of MMP-2 by EGCG. HT1080-cm (A) or purified active MMP-2 (B,C) was pretreated with EDTA before addition of EGCG at indicated concentrations. The mixtures were then analyzed by gelatin zymography (A,B) or FRET-peptide based fluorogenic substrate assay (C). (A) The bar graph is a quantitative representation of pro-MMP-2 activity (in %) relative to control. Bars represent mean±SD (*n*=3). A representative zymogram is shown below. (B) A representative gelatin zymogram showing the effect of EDTA pretreatment on the activity of purified MMP-2. The numbers below the panel indicate relative MMP-2 activity in samples relative to control (set to 1). (C) Result of the FRET-peptide based fluorogenic substrate assay to study the effect of EDTA pretreatment on purified MMP-2 activity. Bars represent mean±SD (*n*=3). (D) EGCG was first pretreated with 3-fold excess of divalent cations. The mixture was then added to purified active MMP-2 followed by analysis of MMP-2 activity by FRET peptide based assay. RFU – relative fluorescence units. *p<0.05, **p<0.01, ***p<0.001.

**Fig. 3:**
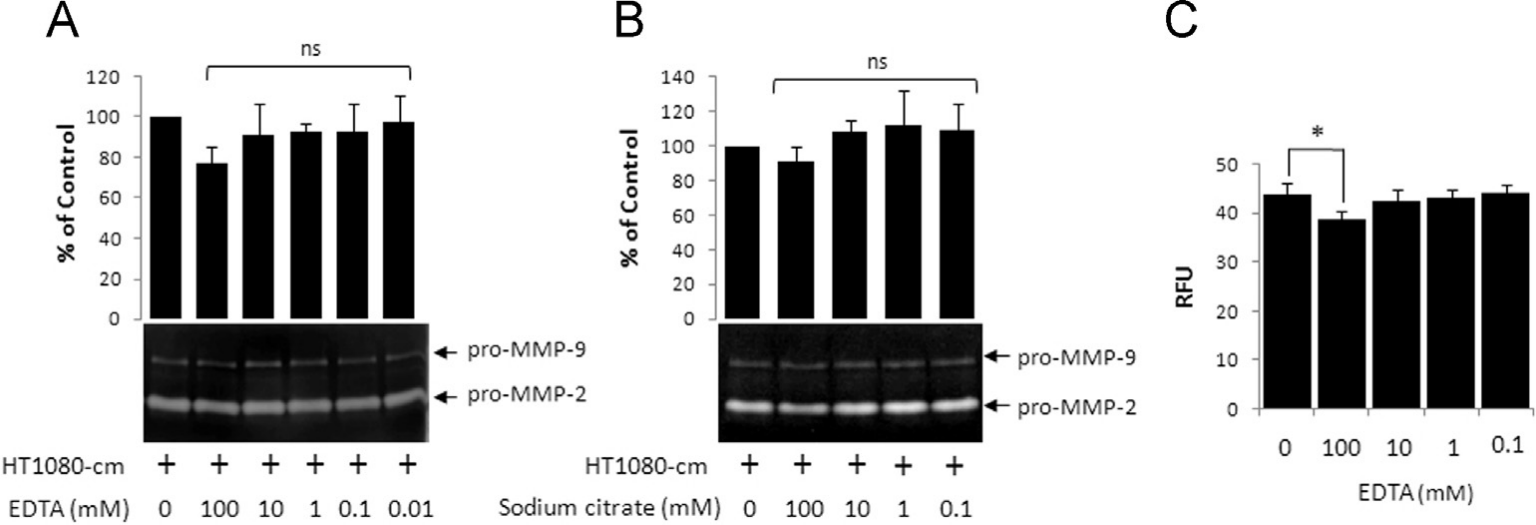
Effect of chelating agents on MMP-2 activity. Aliquots of HT1080-cm were mixed with chelating agents, EDTA (A) or sodium citrate (B), at indicated concentrations and incubated at 37 °C for 30 min. The mixtures were analyzed by gelatin zymography. The background subtracted densitometric measurements for pro-MMP-2 bands in control samples (no EDTA or sodium citrate) were assigned the value of 100 and those obtained in others were expressed as % of control. Bar graphs are results of three independent experiments. (C) Result of the fluorogenic peptide substrate assay for purified active MMP-2 in the presence of indicated concentrations of EDTA. RFU – relative fluorescence units. ns – not significant with respect to control. *p<0.05 (*n*=3).

EGCG inhibited the MMP-2 activity in cell-free assays using HT1080 conditioned medium or the purified active enzyme (Fig. 1). Pretreatment with 10 mM EDTA enhanced EGCG-mediated inhibition of MMP-2 (Fig. 2). Chelating agents alone did not affect MMP-2 activity (Fig. 3).

## 1 Experimental design, materials and methods

### 1.1 Cell culture

HT1080 human fibrosarcoma cells were obtained from the National Centre for Cell Science, Pune, India. They were routinely cultured in 25 cm^2^ flasks (Greiner Bio-one, GmbH, Germany) in a humidified incubator maintained at 37 °C with 5% CO_2_. Cells were fed with phenol red-containing DMEM, which was supplemented with 10% FBS (PAA Laboratories, GmbH, Austria), 100 U/mL penicillin and 100 µg/mL streptomycin (HiMedia, Mumbai, India). When confluent, the HT1080 conditioned medium (HT1080-cm), which is a source of gelatinases namely MMP-2 and MMP-9, was aspirated out and centrifuged at 12,000 rpm for 5 min to remove cell debris. The supernatant (HT1080-cm) was stored in aliquots at −80 °C until use.

### 2.2 Enzymes and reagents

Active human MMP-2 (EC number 3.4.24.24) was purchased from Merck Millipore (Cat. no. PF023). MMP-2 specific fluorogenic peptide substrate, (MCA-Pro-Leu-Ala-Nva-Dpa-Ala-Arg-NH_2_, [MCA=(7-methoxycoumarin-4-yl)acetyl; Dpa=3-(2,4-dinitrophenyl)-L-2,3-diaminopropionyl; Nva=L-norvaline]) was purchased from Merck Millipore (Product no. 444212), dissolved in 100% DMSO to prepare a 10 mM stock and stored in small aliquots at −80 °C. (−)-Epigallocatechin-3-gallate (EGCG) was purchased from Sigma-Aldrich (Product no. E4143, St. Louis, MO, USA) and dissolved in 100% ethanol to prepare a 20 mg/mL (~43 mM) stock.

### 2.3 Experiments to study the effect of EGCG on MMP-2 activity *in vitro*

Aliquots of HT1080-cm were mixed with appropriate volumes of EGCG stock solution to maintain the desired concentrations. Aliquots mixed with equal volume of ethanol alone served as controls. The mixtures were incubated at 37 °C for 45 min and analyzed by gelatin zymography. Alternatively, equal quantities (10 ng) of active human MMP-2 were added to a reaction buffer containing indicated concentrations of EGCG. After incubation at 37 °C for 45 min, the mixtures were analyzed by gelatin zymography. The effect of EGCG on active MMP-2 was also studied using FRET-peptide based fluorogenic substrate assay. This assay is based on cleavage of an MMP-2 specific fluorogenic peptide substrate. In each well of a 96-well plate, 10 ng of active MMP-2 was mixed with indicated concentrations of EGCG in assay buffer (10 mM Tris–HCl at pH 7, 5 mM CaCl_2_, 1 µM ZnCl_2_) in a total volume of 150 µL and incubated at 37 °C for 30 min. This was followed by addition of peptide substrate to a final concentration of 20 µM and further incubation of the reaction mixture at 37 °C for 1 h. Fluorescence was measured using Synergy HT Multimode Microplate reader (BioTeK) using *λ*_max_ excitation of 324 nm and *λ*_max_ emission of 393 nm.

### 2.4 Experiments to study the effect of EDTA and sodium citrate on EGCG-mediated suppression of MMP-2 activity

These experiments were performed exactly as described in the previous section except that HT1080-cm or pure active MMP-2 protein was pre-incubated with indicated concentrations of EDTA or sodium citrate at 37 °C for 30 min prior to addition of EGCG.

### 2.5 Gelatin zymography

Gelatin zymography was performed as described earlier with minor modifications [1]. Samples were resolved in 7.5% SDS-polyacrylamide gels containing 0.1% gelatin under nondenaturing conditions. The gels were washed in 2.5% Triton X-100 to remove SDS and incubated overnight in the activation buffer (10 mM Tris–HCl at pH 7, 5 mM CaCl_2_, 1 µM ZnCl_2_, and 1.5% Triton X-100). The gels were stained with 0.5% coomassie brilliant blue followed by destaining with methanol:glacial acetic acid:water (30:10:60) until clear bands were visible against blue background. The gels were either scanned or photographed using Kodak gel imaging system. The digital images were analyzed by ImageJ software [2]. It should be noted that HT1080-cm samples used in gelatin zymography experiments were not treated with 4-aminophenylmercuric acetate (APMA). Hence the bands detected are of pro-MMP-2 and pro-MMP-9.

### 2.6 Data presentation and analysis

Bar graphs were plotted in MS Excel. Data were analyzed by *R* statistical package [3]. ANOVA and comparison of group means was done using Tukey׳s test at 5% level of significance.
